# An automated framework for understanding structural variations in the binding grooves of MHC class II molecules

**DOI:** 10.1186/1471-2105-11-S1-S55

**Published:** 2010-01-18

**Authors:** Kalidas Yeturu, Tapani Utriainen, Graham JL Kemp, Nagasuma Chandra

**Affiliations:** 1 Bioinformatics Centre, Indian Institute of Science, Bangalore, India; 2 Computer Science and Engineering, Chalmers University of Technology, SE-412 96 Göteborg, Sweden

## Abstract

**Background:**

MHC/HLA class II molecules are important components of the immune system and play a critical role in processes such as phagocytosis. Understanding peptide recognition properties of the hundreds of MHC class II alleles is essential to appreciate determinants of antigenicity and ultimately to predict epitopes. While there are several methods for epitope prediction, each differing in their success rates, there are no reports so far in the literature to systematically characterize the binding sites at the structural level and infer recognition profiles from them.

**Results:**

Here we report a new approach to compare the binding sites of MHC class II molecules using their three dimensional structures. We use a specifically tuned version of our recent algorithm, PocketMatch. We show that our methodology is useful for classification of MHC class II molecules based on similarities or differences among their binding sites. A new module has been used to define binding sites in MHC molecules. Comparison of binding sites of 103 MHC molecules, both at the whole groove and individual sub-pocket levels has been carried out, and their clustering patterns analyzed. While clusters largely agree with serotypic classification, deviations from it and several new insights are obtained from our study. We also present how differences in sub-pockets of molecules associated with a pair of autoimmune diseases, narcolepsy and rheumatoid arthritis, were captured by *PocketMatch*_13_.

**Conclusion:**

The systematic framework for understanding structural variations in MHC class II molecules enables large scale comparison of binding grooves and sub-pockets, which is likely to have direct implications towards predicting epitopes and understanding peptide binding preferences.

## Background

Major histocompatibility complex (MHC) class II molecules are important components of the immune system and play a critical role in processes such as phagocytosis. Antigenic peptide binding by these molecules is a pre-requisite for triggering immune responses. The diversity in antigen recognition is achieved through hundreds of class II alleles labelled by their serotypes, each differing from the others in terms of the residues at the binding site and their precise three dimensional arrangement.

**Figure 1:**
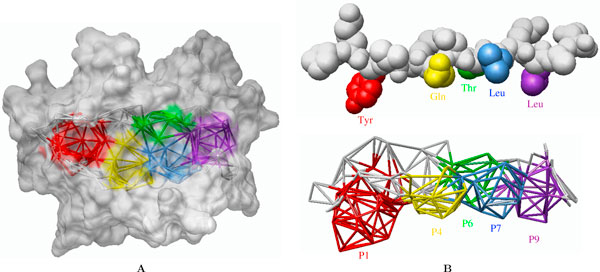
**Structure of an MHC class II binding groove**. **(A) **Binding domain of HLA-DR1 [PDB:1DLH], with the five pockets in the binding groove highlighted (P1 - red; P4 - yellow; P6 - green; P7 - blue; P9 - purple). Lines are drawn between the centres of binding site atoms that can be touched simultaneously by a probe sphere. **(B) **The influenza virus peptide from [PDB:1DLH] is shown above the binding groove, with peptide side chains shown in the same colour as the pockets into which they fit.

The nature of binding site of an MHC class II molecule (Figure 1) has an important bearing on the immune system of an individual [1,2]. MHC class II molecules provide important clues in understanding autoimmune diseases (e.g. [3-5]) and susceptibility to pathogens. In the context of tuberculosis, it has been reported that different MHC alleles bind peptides from *Mycobacterium tuberculosis *with different specificities, influencing an individual's susceptibility to infection [6-8].

A thorough knowledge of the structure of the binding site is useful in designing or identifying peptide antigens for rational vaccine design. In addition, knowledge of similar or dissimilar sites aid in understanding peptide specificities. While a general appreciation of the differences between a pair of structures can be obtained through interactive molecular graphics software tools, a thorough characterization of the differences and their mapping to individual residues in the corresponding structures, and more importantly obtaining a quantitative perspective of the extent of similarities, necessarily requires a systematic method for their analysis.

We have recently reported a new algorithm *PocketMatch *[9] based on alignment of sorted distance elements binned into point-type-pair bins. An important step that precedes pocket comparison is the definition of the binding site itself. In the previous study, all residues (or any atoms in them) that were present in a 4 Å zone around any atom of the ligand were taken to constitute the site. This approach though common, is rather simplistic and more detailed methods to define the binding site need to be explored to have more accurate site definitions. Here we incorporate a new module for defining binding sites and apply it for a large scale comparison of binding sites in the MHC class II molecules.

The modified algorithm is referred to as *PocketMatch*_13 _hereafter. Further, we show that our algorithm is useful for classification of MHC class II molecules based on binding site analysis. The algorithm captures the overall shape, detailed geometry and the chemistry at the binding sites. This analysis also aids in understanding peptide preferences by different alleles which may become the first step in the optimal design of allele specific antigens.

## Results and Discussion

We report a new approach for a large scale comparison of binding sites in protein structures and apply it for comparing and classifying a set of 103 MHC class II molecules. The method, which utilizes structural features of the whole site as well as of the sub-pockets, also serves as a high resolution framework to systematically understand similarities and differences among alleles. We have used this to identify automatically intra- and inter-allelic variations in the binding grooves of molecules in the data set, and to explore the structural basis for correlations with disease.

### Inter-allelic variations

**Figure 2:**
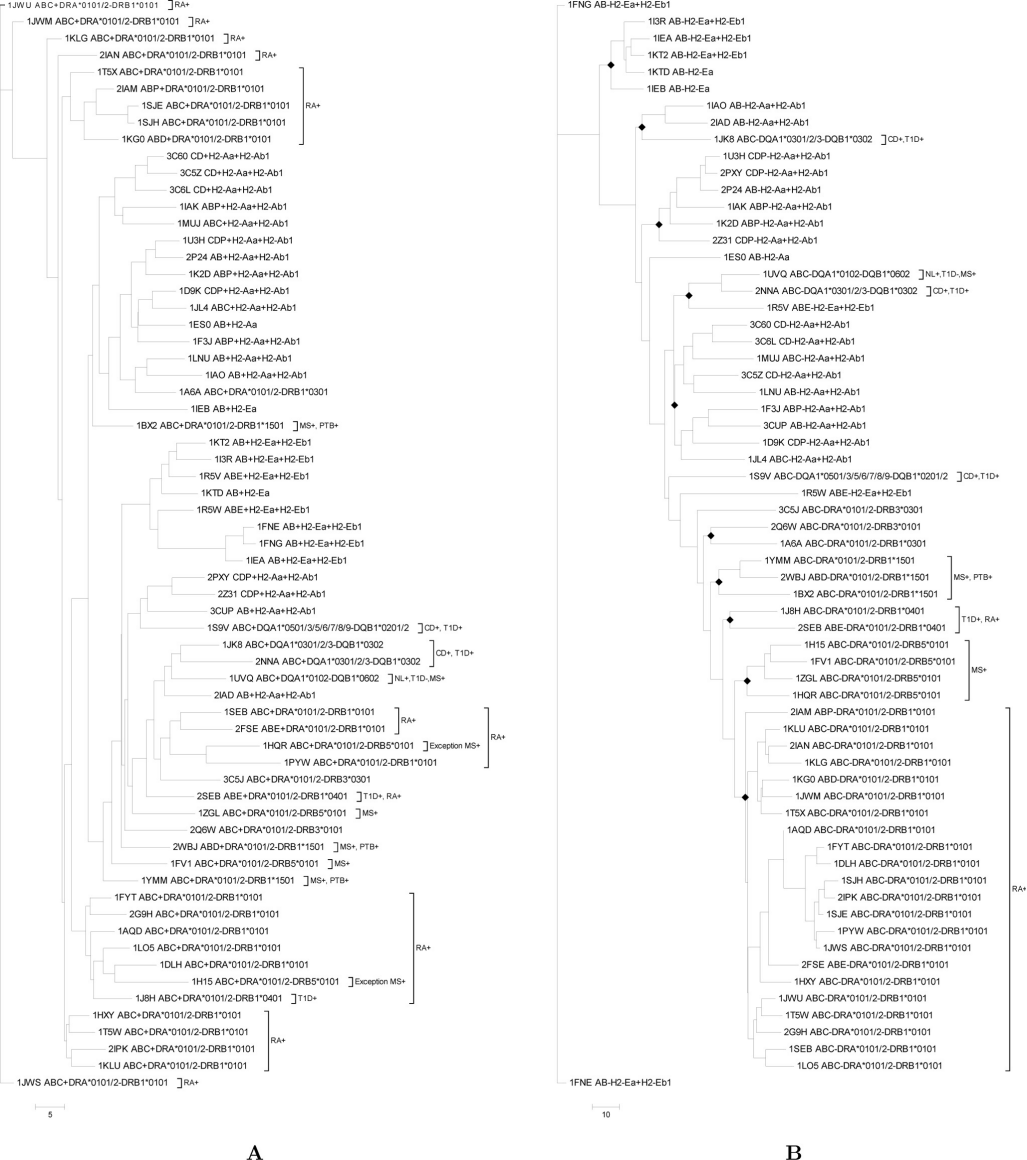
**Cladograms based on similarities between binding sites**. The cladograms for the whole groove and the P4 sub-pocket level similarities among alleles. The first four characters of the label are the PDB identifier for the structure of the MHC class II molecule. The next group of three letters are the chain identifiers used for the alpha chain, beta chain and peptide, respectively, in the original PDB file (there are only two letters in this group in cases where the peptide has been engineered to be part of one of the MHC chains). The final part of the label indicates which alpha and beta alleles are present in the MHC molecule. Branches associated with diseases are shown in *brackets *with disease label. Disease names are abbreviated as NL:Narcolepsy, T1D:Type 1 Diabetes, RA:Rheumatoid Arthritis, CD: Coeliac Disease and PTB:Pulmanory tuberculosis. The suffix '+' stands for positive association and '-' for negative association of an allele with disease. **(A) **The cladogram for the whole groove similarities. **(B) **The cladogram for the P4 similarities. Different branches are indicated by *black diamonds *to indicate net clustering.

To investigate similarities across MHC molecules of different types, one MHC molecule was selected from each of the 65 Protein Data Bank (PDB) entries in the dataset, and all-against-all comparisons were carried out on this set of 65 molecules (Table 1). Binding site similarity scores (*PM*_13_*Scores *) were computed for all the pairs of molecules both at the level of whole groove and sub-pocket levels. Cladograms were generated to show similarities and differences in *PM*_13_*Scores *across the dataset, both at the level of the whole groove, and at the level of the five sub-pockets (Figures 2 and Figures S1-S4 in Additional file 1). In addition to considering whole binding groove, it is important to know how the similarities of the sub-pockets (P1, P4, P6, P7, P9) vary as these are the ones that determine peptide specificity.

**Table 1:** Dataset used in this study. 103 MHC class II molecules from 65 PDB files were used. #Mol -- Number of molecules; #PDB -- Number of PDB entries.

Alleles	**Supertype **[21]	#PDB	PDB Identifiers	#Mol
DQA1*0102-DQB1*0602	DQ1	1	1UVQ	1

DQA1*0301/2/3-DQB1*0302	DQ8	2	1JK8, 2NNA	2

DQA1*0501/3/5/6/7/8/9-DQB1*0201/2	DQ2	1	1S9V	2

DRA*0101/2-DRB1*0101	DR1	22	1AQD, 1DLH, 1FYT, 1HXY, 1JWM, 1JWS, 1JWU, 1KG0, 1KLG, 1KLU, 1LO5, 1PYW, 1SEB, 1SJE, 1SJH, 1T5W, 1T5X, 2FSE, 2G9H, 2IAM, 2IAN, 2IPK	32

DRA*0101/2-DRB1*0301	DR3	1	1A6A	1

DRA*0101/2-DRB1*0401	DR4	2	1J8H, 2SEB	2

DRA*0101/2-DRB1*1501	DR2	3	1BX2, 1YMM, 2WBJ	5

DRA*0101/2-DRB3*0101	DR52	1	2Q6W	2

DRA*0101/2-DRB3*0301	DR52	1	3C5J	1

DRA*0101/2-DRB5*0101	DR51	4	1FV1, 1H15, 1HQR, 1ZGL	8

H2-Aa	--	1	1ES0	1

H2-Aa, H2-Ab1	--	17	1D9K, 1F3J, 1IAK, 1IAO, 1JL4, 1K2D, 1LNU, 1MUJ, 1U3H, 2IAD, 2P24, 2PXY, 2Z31, 3C5Z, 3C60, 3C6L, 3CUP	26

H2-Ea	--	2	1IEB, 1KTD	4

H2-Ea, H2-Eb1	--	7	1FNE, 1FNG, 1I3R, 1IEA, 1KT2, 1R5V, 1R5W	16

Some MHC molecules of the same type are in different branches of the cladogram calculated for the whole groove, however clustering at the sub-pocket level was more in line with the different MHC molecule types, particularly for the P4 sub-pocket. This suggests that the P4 sub-pocket is more structurally conserved within an allele, but difference occurs across alleles. The importance of the P4 sub-pocket has been noted in many studies (e.g. [1,2,10]).

Some different MHC molecules are grouped together in the same branch in some of the sub-pocket trees. In these cases, the *PM*_13_*Scores *highlight similarities that would otherwise be difficult to spot in a large dataset. These can be followed up by looking for independent observations about these similarities that have been reported in the literature. The matching alleles, corresponding PDB codes and *PM*_13_*Scores *for pairs of sub-pockets are listed in (Table 2), where the significance of the grouping of different alleles is discussed and supporting references are presented.

**Table 2:** Summary of the analysis performed on cladograms for the whole groove and five sub-pockets. For a pair of different alleles, the pair of molecules obtaining high *PM*_13 _*Score *on whole groove or sub-pocket comparison is presented. Literature citation for other independent work supporting the observation is also provided wherever possible. The examples shown here refer to different alleles that appear in the same branch of the clustergarm computed either by using the whole grooves or by their individual sub-pockets as indicated in each row.

Pair of alleles	(PDB1, PDB2)	Pocket	***PM***_**13**_ ***Score***	Comment
DQB1*0602-DQB1*0302	(1UVQ, 1JK8)	whole	0.83	The involvement of the two alleles DQB1*0602 and DQB1*0302, negatively and positively associated with Type 1 diabetes is reported by Siebold and co-workers [22]
	(1UVQ, 2NNA)		0.75	
	(1UVQ, 2NNA)	P4	0.74	
	(1UVQ, 1JK8)	P7	0.71	

DQB1*0201, 2-DRB1*1501	(1S9V, 1BX2)	P7	0.4	
DQB1*0201, 2-DRB1*1501	(1S9V, 1BX2)	P9	0.08	

DRB1*0401-DRB1*0101	(2SEB, 2FSE)	P9	0.61	
	(1J8H, 2IPK)		0.85	The study by Rosloniec and co-workers [13] indicate the association of the two alleles are known to be associated with RA.

DRB1*1501-DRB1*0101	(2WBJ, 1LO5)	P9	0.69	These observations agree with the study by Smith and co-workers [23] that reports the similarity of the P9 sub-pocket. Study by Drouin and co-workers [24] refer to the association of the two alleles with antibiotic-refractory arthritis.
	(1YMM, 1KLU)		0.59	
	(1YMM, 2IAM)		0.66	

DRB1*1501-DRB1*0301	(1BX2, 1A6A)	whole	0.80	Both alleles came in two branches under a common root which is in accordance with a study by Zivadinov and co-workers [25] that associates the two alleles to Multiple sclerosis.

DRB3*0101-DRB1*0101	(2Q6W, 1HXY)	P1	0.66	

DRB3*0101-DRB1*0301	(2Q6W, 1A6A)	P4	0.54	The two molecules are grouped together. Though the score is only 0.54, there are no other molecules they could come similar to with matching allele types. The study by Parry and co-workers [26] indicate the expected similarity in the P4 sub-pocket and correlate the differences in other subpockets and the differences in P4 itself to difference between the two alleles in susceptibility to Type 1 diabetes.

DRB3*0301-DRB1*0401	(3C5J, 1J8H)	P6	0.43	
	(3C5J, 2SEB)		0.37	
	(3C5J, 2SEB)	P7	0.77	

DRB5*0101-DRB1*0101	(1HQR, 1PYW)	whole	0.78	
	(1HQR, 2G9H)	P1	0.9	Meinl and co-workers [27] also report similarity between the two allele types in recognition of myelin basic protein. The P1 similarity between the two alleles is reported by Jurcevic and co-workers [28].
	(1H15, 1DLH)	whole	0.8	
	(1H15, 1LO5)	P1	0.73	
	(1ZGL, 1JWM)	P1	0.8	

DRB5*0101-DRB1*0301	(1FV1, 1A6A)	P1	0.81	Though P1 score is high, the other subpocket scores are low (less than 0.34) which is in accordance with study by Texier and co-workers [29] that reports difference between the two alleles in their peptide binding properties.
		P9	0.56	
	(1FV1, 1R5W)	P9	0.6	
	(2Q6W, 3C5J)	P9	0.84	This similarity of P9 is a known feature [30] for the two, DR52a and DR52c alleles which are encoded by the DR3 gene whose alleles are all associated with autoimmune diseases.

DQB1*0602-H2-Aa, H2-Ab1	(1UVQ, 1IAK)	P6	0.64	Orthologous alleles from human and mouse [31].
	(1UVQ, 1JL4)	P9	0.71	
	(1UVQ, 2IAD)	P9	0.75	

DQB1*0201-H2-Aa, H2-Ab1	(1S9V, 2PXY)	whole	0.73	
	(1S9V, 2Z31)		0.79	
	(1S9V, 3CUP)		0.78	
	(1S9V, 1MUJ)	P1	0.57	

DRB1*0101-H2-Aa, H2-Ab1	(1AQD, 1K2D)	P1	0.52	
	(1D9K, 1SJE)	P9	0.46	
	(1D9K, 1SJH)	P9	0.53	

DRB1*0301-H2-Aa, H2-Ab1	(1A6A, 1LNU)	whole	0.83	
	(1A6A, 1IAO)		0.83	

DRB1*1501-H2-Aa, H2-Ab1	(2WBJ, 1IAO)	P7	0.42	
	(2WBJ, 1LNU)		0.57	

DRB1*1501-H2-Ea, H2-Eb1	(1R5V, 1BX2)	P6	0.65	Orthologous alleles from human and mouse.

**Figure 3:**
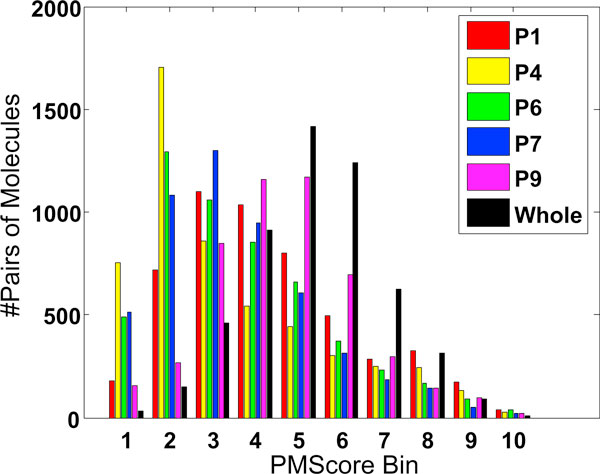
**Frequencies of *PM*_13_*Scores *(labelled PMScore) for the five sub-pockets and whole binding groove**. X-axis: 10 bins of *PM*_13_*Scores *ranging from 0 to 1.0. Red, yellow, green, blue and purple bars correspond to the P1, P4, P6, P7 and P9 sub-pockets. Black bars correspond to whole groove similarity scores.

To analyze the net distribution of similarity scores with respect to each other for each of the five sub-pockets, a histogram is plotted for various bins of *PM*_13_*Scores *(Figure 3). Each bin corresponds to a range of *PM*_13_*Scores*. For example, bin-5 corresponds to a *PM*_13_*Score *range of [0.5 to 0.6); bin-7 to the range [0.7 to 0.8) and so on. The histogram shows that P1 and P9 score highly at bin 6, corresponding to [0.6 to 0.7) of *PM*_13_*Score*. The histogram gives an indication of the overall distribution of scores for each sub-pocket viewed in the context of others. This could possibly mean over-representation of data or true conservation of these two sub-pockets.

This analysis has implications for understanding subtle differences that otherwise go undetected and aid in understanding antigen recognition preferences by different alleles and range of antigens recognized by a given allele.

### Intra-allelic variations

Some MHC molecules are present more than once in the PDB entries in the dataset (Table 1). In these cases, *PocketMatch*_13 _can be used to highlight differences in the peptide binding sites in different structures for the same allele.

**Figure 4:**
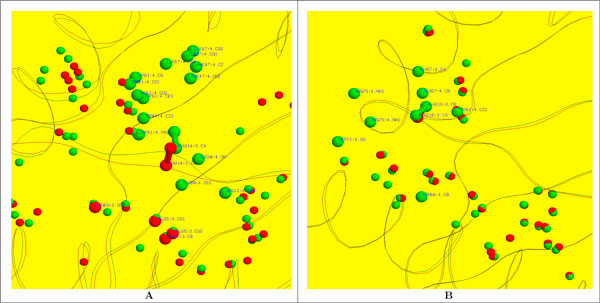
**Examples of sub-pockets with low *PM*_13 _*Scores***. **(A) **Detail of the superposed binding grooves of [PDB:1BX2] (red) and [PDB:2WBJ] (green). MHC and peptide main chains are represented by a cartoon trace. Spheres indicate the centres of MHC atoms that are determined to be part of the binding groove (see Methods). The centres of MHC atoms that are determined to be part of the P7 sub-pocket are represented by the larger spheres; these atoms are labelled in blue. The C*α *and C*β *atoms of the peptide residues at the P7 positions, remodelled as alanines, are shown with a ball-and-stick representation. **(B) **Similar to (A), but focusing on the P9 sub-pockets in [PDB:1S9V] (chains A, B, C in red; chains D, E, F in green).

The sites are first compared by considering the whole binding grooves. In many cases, as expected, *PM*_13_*Scores *are high, indicating strong similarities in the binding sites of a given allele. However, there are cases where *PM*_13_*Scores *are low for different structures of the same molecule, for example different structures of DR1 and DR5 give similarity scores as low as 0.44 (Table S1 in Additional file 1). These differences can be explored by examining the individual sub-pockets within the binding grooves (see Methods). While many pairs of corresponding sub-pockets score highly, indicating similarity in the structures of the sub-pockets, in some cases the scores are significantly lower. This can be due to differences in MHC side chain conformations giving rise to different sets of intra-site distances, or can be due to determination of which MHC atoms are accessible to a probe sphere and are thus included in sub-pocket calculations. Sub-pockets highlighted by *PocketMatch*_13 _to be dissimilar can then be examined in detail to identify the reason for the low *PM*_13_*Scores*. Some examples of sub-pockets with low *PM*_13 _*Scores *are illustrated in Figure 4.

A pair of molecules belonging to DR1 exhibited low scores [PDB:1AQD, PDB:1DLH] in their P1 sub-pockets. Upon careful examination, we noticed that the P1 sub-pocket in 1DLH was wider and deeper with many more MHC atoms being included in the *PocketMatch*_13 _definition of the P1 sub-pocket. Considering the set of DRA*0101-DRB1*1501 structures, the largest difference is between the P7 pockets of [PDB:1BX2] and [PDB:2WBJ] (Figure 4A). The peptide residue at the P7 position is oriented very differently in these two structures -- in [PDB:1BX2], an isoleucine is oriented away from the groove, whereas in [PDB:2WBJ] a leucine is oriented "across" the top of the groove. Since the P7 peptide residue in [PDB:2WBJ] obstructs the P7 sub-pocket more than the P7 peptide residue in [PDB:1BX2], this affects the set of MHC atoms that are selected for the sub-pocket comparison calculation, and thus reduces the *PM*_13_*Score *(0.06).

The two independent molecules in the crystal structure of DQ8 [PDB:1S9V] differ from each other at the P9 sub-pocket (Figure 4B); the difference between the two molecules at the P9 position is noted by [11]. This analysis indicates that *PocketMatch*_13 _is sufficiently sensitive to capture subtle differences that exist among molecules belonging to the same allele.

### Correlation with disease: case studies

**Figure 5:**
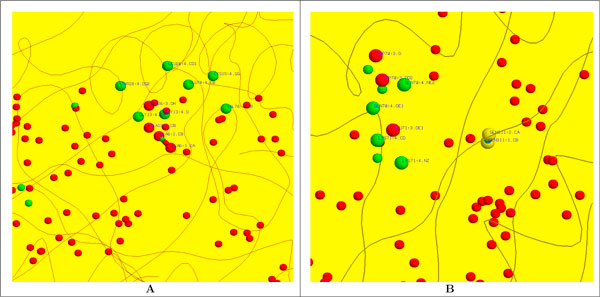
**Detail of the binding grooves of HLA-DQ6.2 and HLA-DR4.2**. **(A) **Detail of the binding groove of HLA-DQ6.2 (green). MHC and peptide main chains are represented by a cartoon trace. Spheres indicate the centres of MHC atoms that are determined to be part of the binding groove (see Methods). The centres of MHC atoms that are determined to be part of the P4 sub-pocket are represented by the larger spheres; these atoms are labelled in blue. The C*α *and C*β *atoms of the peptide residue at the P7 position, remodelled as alanine, is shown with a ball-and-stick representation. Atoms that differ in binding groove of the model structure of HLA-DQ6.1 are shown in red. **(B) **Detail of the binding groove of HLA-DR4.2 (red). MHC and peptide main chains are represented by a cartoon trace. Spheres indicate the centres of MHC atoms that are determined to be part of the binding groove (see Methods). Atoms that differ in binding groove of the model structure of HLA-DR4.1 are shown in green. The centres of atoms of residues 70*β *and 71*β *are represented by the larger spheres; these atoms are labelled in blue. The C*α *and C*β *atoms of the glutamine residue at the P4 position in the peptide are shown with a ball-and-stick representation (yellow).

Several MHC class II alleles are known to be either positively or negatively associated with certain diseases, and this motivates studies to identify the reasons for disease susceptibility in terms of three-dimensional molecular structure [1]. For example, Jones *et al*. [1] review the structures of alleles that are known to be positively or negatively associated with various diseases, including narcolepsy and rheumatoid arthritis (RA). We have used *PocketMatch*_13 _to examine the binding grooves of alleles discussed by Jones *et al*. [1] in connection with narcolepsy and RA, using experimentally determined structures from the PDB where these are available, and model structures when they are not (see Methods). In case of Narcolepsy, the pockets of the binding groove in the experimentally determined structure of HLA-DQ6.2 (positively associated with the disease) [PDB:1UVQ], were compared to those in a model structure of HLA-DQ6.1 (negatively associated with the disease). These molecules differ at only a few positions in the *β *chain. *PocketMatch*_13 _identified the P4 sub-pocket corresponding to the Thr6 residue of the peptide to be the most dissimilar between these two structures (Table 3). The residues Ala13b*β *and Tyr26*β *in HLA-DQ6.2 changed to Gly13*β *and Leu26*β *in HLA-DQ6.1 in the neighbourhood of peptide residue Thr6, corresponding to P4 (Figure 5A); this difference is captured by the *PocketMatch*_13 _algorithm.

**Table 3:** Similarity scores between sub-pockets of HLA-DQ6.1 and HLA-DQ6.2. PMSMax and PMSMin are defined in Methods.

Peptide residue	Pocket	PMSMin	PMSMax
Leu3	P1	1.0	1.0
Thr6	P4	0.57	0.84
Val8	P6	0.90	0.90
Ser9	P7	1.0	1.0
Ala11	P9	0.92	0.92

In case of RA, alleles HLA-DR4.1, HLA-DR4.4 and HLA-DR1 are positively associated with the disease, while HLA-DR4.2 is neutral or negative [1]. The *α *chains of these four MHC molecules are the same (DRA*0101), and sequence comparison of the *β *chains with ClustalW [12] gives sequence identities of -- DR4.1:DR4.2 = 95%, DR4.1:DR4.4 = 97%, DR4.1:DR1 = 88%, DR4.2:DR4.4 = 96%, DR4.2:DR1 = 85%, DR4.4:DR1 = 88%. Given that the whole sequence similarities are not sensitive enough to capture differences at the binding site levels, we use *PocketMatch*_13 _to compare the binding grooves and sub-pockets of the experimentally determined structures of HLA-DR4.1 [PDB:1J8H] and HLA-DR1 [PDB:1DLH], and model structures of HLA-DR4.2 and HLA-DR4.4.

*PocketMatch*_13 _gives low scores for the P4 sub-pocket (Table 4A). It has been shown by Hammer and co-workers [10] that the difference in residues 70 and 71 in the *β *chain of the DR4.1 and DR4.2 MHCs accounts for the difference in binding specificity of the peptides. The low P4 scores are in line with that study. The superposition of these two alleles is shown in Figure 5B. The P4 peptide residue has Gln70*β *and Lys71*β *present in HLA-DR4.1 within 3.0 Å of the residue whereas an Asp at the position 70*β *and only Glu71*β *are present in the case of the model built for HLA-DR4.2.

**Table 4:** Sub-pocket similarities. (A) Sub-pocket similarities between a pair of alleles HLA-DR4.1 [PDB:1J8H] and HLA-DR4.2 (model) are shown. The residues of the peptide are shown on the left most column. Residue numbers 311 and 314 correspond to P4 and P7 respectively. The low *PM*_13_*Scores *are shown in boldface. (B) Variation of the P4 similarity scores among HLA-DRB1*0101 [PDB:1DLH], HLA-DR4.1 [PDB:1J8H], HLA-DR4.4 and HLA-DR4.2 are shown. (C) Variation in P7 similarities are shown for proteins mentioned under (B).

(A) - HLA-DR4.1(1J8H-abc) and HLA-DR4.2(model)			
**Pocket**	**Residue**	**PMSMin**	**PMSMax**

P1	Tyr308	0.85	0.91
P4	Gln311	0.79	0.85
P6	Thr313	0.89	0.89
P7	Leu314	0.53	0.71
P9	Leu316	1.0	1.0

**(B) - P4 (Gln311)-similarity**			

**PDB/MODEL**	**PDB/MODEL**	**PMSMin**	**PMSMax**

1DLH	1J8H	0.56	0.60
1DLH	HLA-DR4.2	0.53	0.53
1DLH	HLA-DR4.4	0.63	0.68
1J8H	HLA-DR4.2	0.79	0.85
1J8H	HLA-DR4.4	0.86	0.86
HLA-DR4.2	HLA-DR4.4	0.74	0.79

**(C) - P7 (Leu314)-similarity**			

**PDB/MODEL**	**PDB/MODEL**	**PMSMin**	**PMSMax**

1DLH	1J8H	0.44	0.58
1DLH	HLA-DR4.2	0.50	0.50
1DLH	HLA-DR4.4	0.67	0.67
1J8H	HLA-DR4.2	0.53	0.71
1J8H	HLA-DR4.4	0.60	0.81
HLA-DR4.2	HLA-DR4.4	0.57	0.57

All-against-all *PM*_13_*Scores *are presented in Table 4B, C. The scores indicate low *PM*_13_*Score *of [PDB:1DLH] to others in the P7 region of the binding site. Work by Rosloniec and co-workers found that mutation of the residue at the P7 position to an alanine has affected T cell stimulation more with DR4 than with DR1 [13]. The involvement of P7 sub-pocket in peptide recognition specificity is also discussed in [10]. In carrying out these case studies, model structures have been a useful supplement to the set of experimentally determined MHC class II molecules. We envisage future studies that make use of larger sets of model structures where the binding grooves have been modelled consistently using the same protocol [14].

## Conclusion

A strategy for automatically comparing MHC class II binding grooves and sub-pockets based on their chemical nature and geometry is presented. Comparisons are facilitated by a pre-processing step in which MHC-peptide complexes are extracted from PDB files, and chains and structurally equivalent residue positions are relabelled consistently. Pocket similarity scores calculated by *PocketMatch*_13 _can be used as the basis for clustering pockets based on their structural and chemical characteristics.

The framework we report can be used to carry out large scale comparison of binding grooves and sub-pockets, both to highlight differences in the binding grooves of MHC molecules of the same kind, and to identify similarities in the binding grooves of different MHC alleles. Investigations of MHC alleles associated with narcolepsy and rheumatoid arthritis demonstrate that binding grooves of alleles that are positively associated with an autoimmune disease can be compared with those that are known to be negatively associated with the disease. The structural variations among binding pockets identified by *PocketMatch*_13 _corroborate known disease associations. Future applications of this systematic framework for understanding structural variations in MHC class II molecules could have direct implications towards predicting epitopes and understanding peptide binding preferences.

## Methods

### Dataset preparation

103 MHC class II molecules from 65 Protein Data Bank [15] entries are used in this study (Table 1), and the sequences of the *α*_1 _and *β*_1 _domains from these structures were matched with allele sequences from IMGT/HLA database [16] to confirm which allele is present in the PDB entry. In this study, the focus is on MHC class II binding domains. In some cases, different alleles share identical sequences for the binding region, e.g. human alpha chains DRA*0101 and DRA*0102 have binding domains with identical sequences, so both of these alleles are listed alongside structures with this alpha chain sequence in Table 1. Similarly, many alleles have binding domains with sequences that are identical to those in [PDB:1S9V], and these are listed in Table 1.

To facilitate automatic comparison of MHC class II structures, uniform chain identifiers and residue numbers were used for all MHC-peptide complexes extracted from the PDB files. New files were written where each file contains the core parts of an *α*_1 _domain, a *β*_1 _and a peptide, with chains relabelled to match the chain identifiers A, B and C in [PDB:1DLH], and residues renumbered to match the numbering of residues at structurally equivalent positions in [PDB:1DLH]. Positions 5-78 of the *α*_1 _domain and positions 5-91 of the *β*_1 _domain were retained. A rigid body transformation was applied to superpose the the MHC binding domain complexes onto chains A and B of [PDB:1DLH<http://www.rcsb.org/pdb/cgi/explore.cgi?pdbId=1DLH>], so that all complexes are in the same frame of reference. This transformation is not necessary for the automatic comparisons that follow, but it is convenient for comparing structures using molecular graphics to review results from the automatic comparisons. Peptide residues corresponding to the 13 peptide residues in [PDB:1DLH] were identified by structural comparison, and peptide residues beyond the 13-residue peptide present in [PDB:1DLH] were removed automatically.

### Comparative modelling

To enable the comparison of binding grooves of MHC class II molecules known to be positively or negatively associated with narcolepsy or RA, models of HLA-DQ6.1 consisting of alleles (HLA-DQA1*0102 and HLA-DQB1*0601), HLA-DRB4.2 (alleles HLA-DRA1*0101 and HLA-DRB1*0402) and HLA-DRB4.4 (alleles HLA-DRA1*0101 and HLA-DRB1*0404) were built interactively using the Swiss-PdbViewer [17]. [PDB:1UVQ] was used as the template structure for the model of HLA-DQ6.1 and [PDB:1J8H] was used as the template for HLA-DRB4.2 and HLA-DRB4.4.

### Binding site comparison

Binding sites are represented in a frame invariant manner by distances between pairs of points, partitioned into bins, and pairs of sites are compared based on alignment of sorted sequences of distances. The sorted arrays are then aligned and scored to finally obtain comparison scores.

Molecules can be clustered based on their comparison scores.

In this study, the points used are the centres of those atoms lining the binding site. These are determined by considering accessibility to a probe sphere with radius 1.4 Å. Those MHC atoms whose accessibility is reduced by the presence of the peptide are determined to be part of the peptide binding site. Similarly, the MHC atoms that comprise individual pockets are identified as the set of atoms whose accessibility is reduced by the presence of the peptide residue at position P1, P4, P6, P7 or P9. The ProtOr radii from Table 2 of [18] are used for protein atomic groups in accessibility calculations.

The corresponding pockets between a pair of MHC binding sites are compared on large scale in an all-against-all comparison scheme. The shape signature of each pocket, capturing chemical nature and geometric distribution of atoms, is derived based on the distance lists concept used in *PocketMatch *[9].

Site comparison proceeds as follows:

• Surface atomic groups are classified into 13 types based on heavy-atom types, the number of covalently attached hydrogen atoms and the number of all covalently attached atoms, as proposed by Tsai et al. [18]: C3H0, C3H1, C4H1, C4H2, C4H3, N3H0, N3H1, N3H2, N4H3, O1H0, O2H1, S2H0, S2H1.

• Distances between all pairs of atoms are computed and binned into 13 * (13 - 1)/2 + 13 → 91 lists corresponding to each pair of atomic types (C3H0-C3H0, C3H0-C3H1, etc.)

• Each list or bin of distances is then sorted in non-decreasing order. The sorted distance elements binned into various lists according to chemical nature of the atoms constitutes the shape descriptor of the binding pocket.

• To compare a pair of sites, each of the 91 lists is chosen in one site together with the corresponding list from the other site, and the cumulative number of similar distance elements is determined.

• A pair of distances from two lists is marked a match if the distance differ at most by a threshold of 0.5.

We call the tuned version of *PocketMatch *for the MHC class II binding site comparison, by considering solvent accessible atoms and 13 atomic group types, *PocketMatch*_13_.

The numerator is simply the number of matching intra-site distances. However, the denominator can be the number of intra-site distances in either the smaller site or in the larger site -- these give rise to two *PM*_13_*Score *values, referred to as PMSMax and PMSMin, respectively. Unless stated otherwise, *PM*_13_*Score *refers to the PMSMin value.

**Figure 6:**
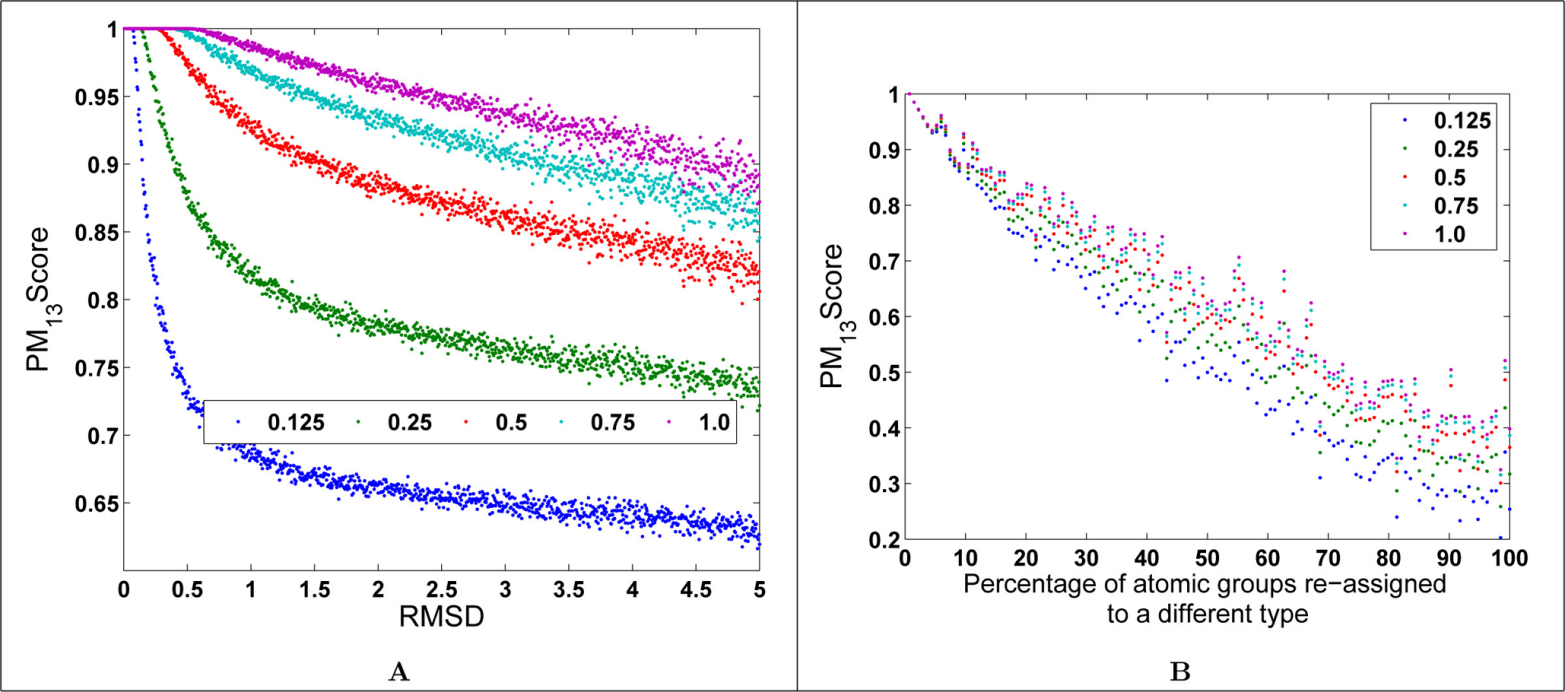
**Effect of altering the geometry or chemistry of the binding groove on *PM*_13_*Scores***. Altered binding grooves are compared with the original using *PocketMatch*_13_. *PM*_13_*Scores *calculated with different distance element alignment thresholds are shown in different colours (1.0 Å in purple; 0.75 Å in cyan; 0.5 Å in red; 0.25 Å in green; 0.125 Å in blue). (A) The coordinates of atoms in the binding groove of [PDB:1JWS] (A, B, C chains) were perturbed randomly, and an ensemble of 1000 structures was generated with RMSD values up to 5 Å with respect to the original [PDB:1JWS] structure. (B) The atomic group labels of some of the atomic groups in the binding groove of [PDB:1JWS] (A, B, C chains) were re-assigned randomly.

*PM*_13_*Score *values decrease as the similarity between a pair of binding grooves decreases (Figure 6). The rate at which the scores decrease is affected by the threshold chosen for site comparison, since this affects the number of matching distance elements between a pair of distance-sequences. To illustrate the effect of perturbing the conformation of a binding groove, the coordinates of atoms in the binding groove of [PDB:1JWS] (A, B, C chains) were perturbed randomly, and an ensemble of 1000 structures was generated with root mean square deviation (RMSD) values up to 5 Å with respect to the original [PDB:1JWS] structure. We have used a similar strategy for sensitivity analysis for the original *PocketMatch *algorithm [9] and found that a threshold of 0.5 Å was adequate to distinguish between similar and dissimilar sites. Figure 6A shows the *PM*_13_*Scores *obtained by comparing the original [PDB:1JWS] structure with each of the perturbed structures in the ensemble. Rather than perturbing the atomic coordinates randomly, an alternative method for generating an ensemble of perturbed conformations would be to use conformations from a molecular dynamics trajectory. To investigate the effect of altering the chemical nature of the binding groove while retaining its original geometry, the atomic group labels of some of the atomic groups in the binding groove of [PDB:1JWS] (A, B, C chains) were re-assigned randomly, and *PocketMatch*_13 _was used to compare the modified binding groove with the original one (Figure 6B). Figures 6A and 6B demonstrate that *PM*_13_*Scores *capture differences due to both the geometry and the chemical nature of the binding groove.

### Cladogram generation

Given a set of binding sites (whole groove or sub-pockets), one way of visualizing the relationships among these is to generate a cladogram based on distances between pairs of sites. The distance between a pair of sites is defined here to be 1-*PM*_13_*Score *between the two sites. The cladogram generation program is based on the *neighbour joining *method available in Phylip-3.67 [19] which generates trees in Newick format, which can be visualized and labelled using MEGA [20]. When generating cladograms, data were input to the program in descending order of *PM*_13_*Scores*.

## Competing interests

The authors declare that they have no competing interests.

## Authors' contributions

KY participated in implementation of the atom type version of PocketMatch, setting up of computational framework for large scale site comparisons and helped to draft the manuscript. TU participated in preparing the data set. GJLK participated in the design and coordination of the study and helped to draft the manuscript. NC participated in reviewing results, manuscript and scientific discussions.

## Supplementary Material

Additional File 1 A zip compressed archive with supplementary Figures S1-4 and Table S1. Click here for file
