# Supplementary material for: An automated framework for understanding structural variations in the binding grooves of MHC class II molecules
Source: BMC Bioinformatics. 2010 Jan 18;11(Suppl 1):S55. doi: 10.1186/1471-2105-11-S1-S55 (PMC3009528; doi:10.1186/1471-2105-11-S1-S55)
Supplement: Additional File 1 — A zip compressed archive with supplementary Figures S1-4 and Table S1. [file 1471-2105-11-S1-S55-S1.zip › Table-S1.pdf]

**Allele**	**#Molecules**	**Whole**	**P1**	**P4**	**P6**	**P7**	**P9**
DQA1*0301/2/3-DQB1*0302	2	0.80, 0.80	0.82, 0.82	0.49, 0.49	0.82, 0.82	0.62, 0.62	0.68, 0.68
H2-Aa	1	X,X	X,X	X,X	X,X	X,X	X,X
DRA*0101/2-DRB1*1501	5	0.73, 0.88	0.50, 0.92	0.50, 0.80	0.49, 0.91	**0.06**, 0.79	**0.07**, 0.72
DRA*0101/2-DRB1*0401	2	0.70, 0.70	0.67, 0.67	0.58, 0.58	0.61, 0.61	0.70, 0.70	0.25, 0.25
DQA1*0102-DQB1*0602	1	X,X	X,X	X,X	X,X	X,X	X,X
DQA1*0501/3/5/6/7/8/9-DQB1*0201/2	2	0.70, 0.70	0.90, 0.90	0.84, 0.84	0.89, 0.89	0.68, 0.68	**0.27**, 0.27
DRA*0101/2-DRB3*0301	1	X,X	X,X	X,X	X,X	X,X	X,X
H2-Ea	4	0.70, 0.90	0.61, 0.84	0.17, 0.76	0.83, 0.95	0.49, 0.88	0.60, 0.80
DRA*0101/2-DRB1*0301	1	X,X	X,X	X,X	X,X	X,X	X,X
H2-Aa,H2-Ab1	26	0.59, 0.99	***0.19**,1	***0.18**,1	***0.15**,1	***0.22**,1	***0.17**,1
DRA*0101/2-DRB3*0101	2	0.81, 0.81	0.82, 0.82	0.66, 0.66	0.71, 0.71	0.67, 0.67	0.67, 0.67
H2-Ea,H2-Eb1	16	0.72, 0.97	0.52, 1	***0.12**,0.94	***0.16**,1	***0.22**,1	***0.30**,1
DRA*0101/2-DRB1*0101	32	0.44, 1	***0.09**,1	0.49, 1	***0.25**,1	***0.20**,1	***0.18**,1
DRA*0101/2-DRB5*0101	8	0.47, 0.96	***0.21**,0.89	0.60, 0.85	***0.04**,0.90	***0**,1	***0.31**,0.89
