# Supplementary figures and images for: An automated framework for understanding structural variations in the binding grooves of MHC class II molecules
Source: BMC Bioinformatics. 2010 Jan 18;11(Suppl 1):S55. doi: 10.1186/1471-2105-11-S1-S55 (PMC3009528; doi:10.1186/1471-2105-11-S1-S55)

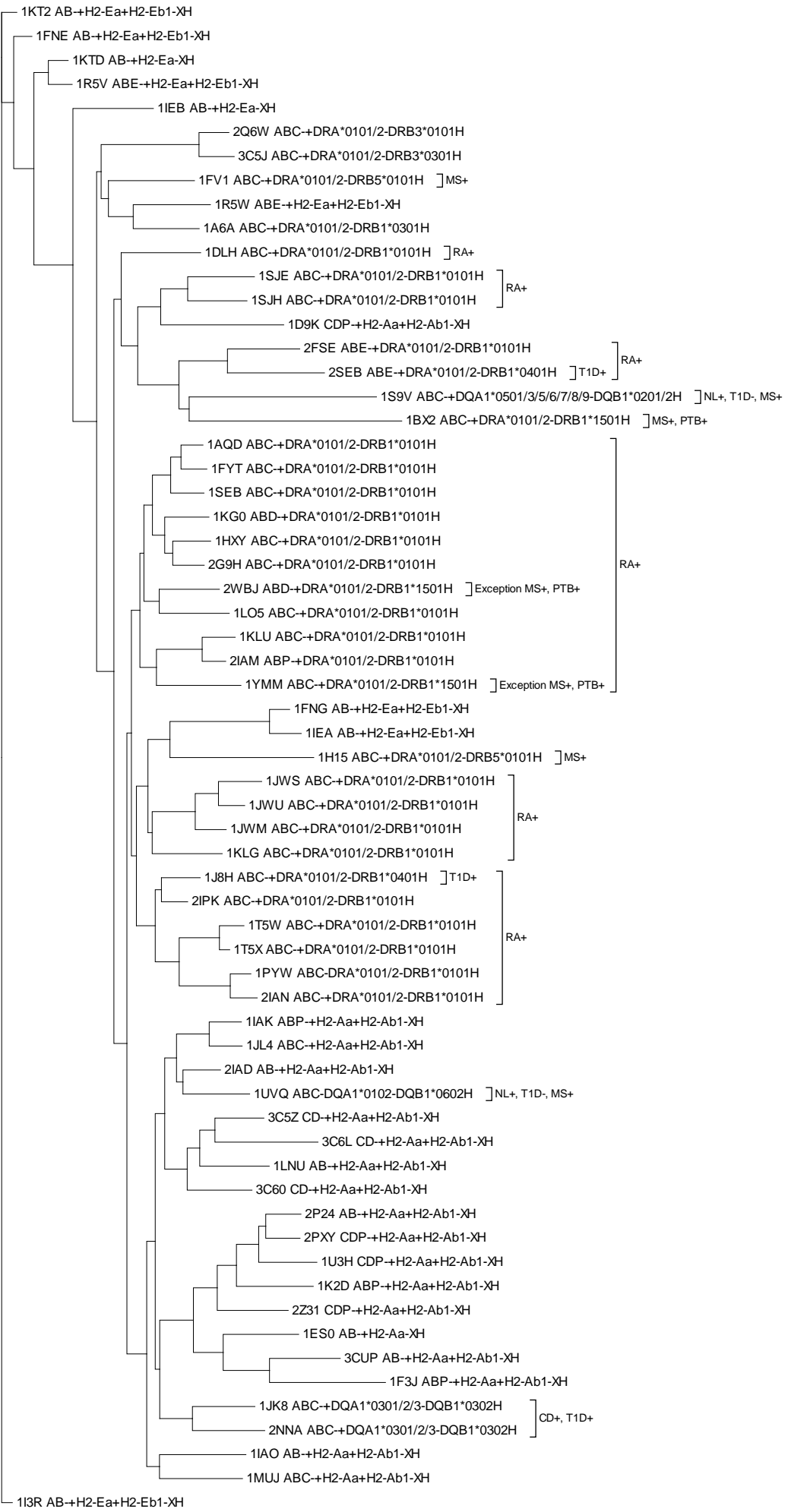

Supplement: Additional File 1 — A zip compressed archive with supplementary Figures S1-4 and Table S1. [file 1471-2105-11-S1-S55-S1.zip › Figure-S4.pdf]

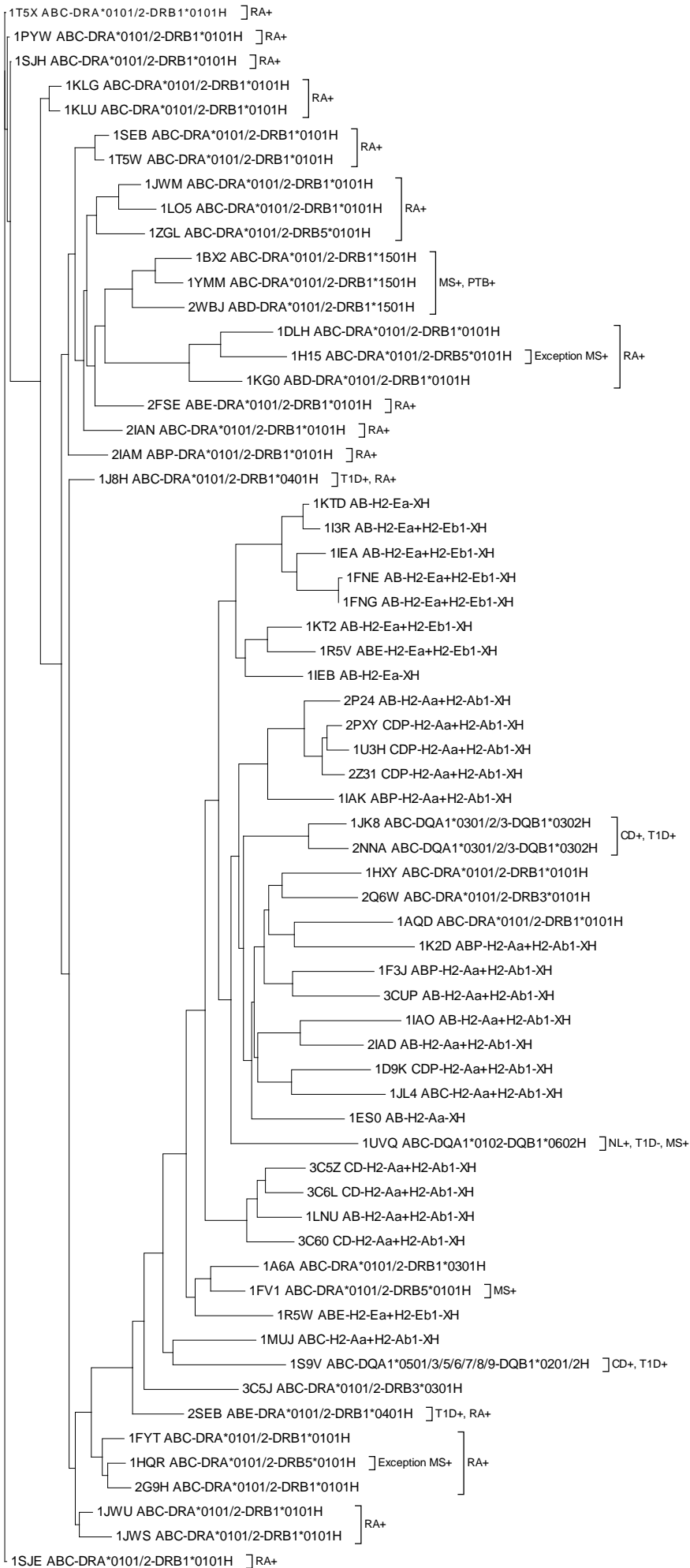

Supplement: Additional File 1 — A zip compressed archive with supplementary Figures S1-4 and Table S1. [file 1471-2105-11-S1-S55-S1.zip › Figure-S1.pdf]

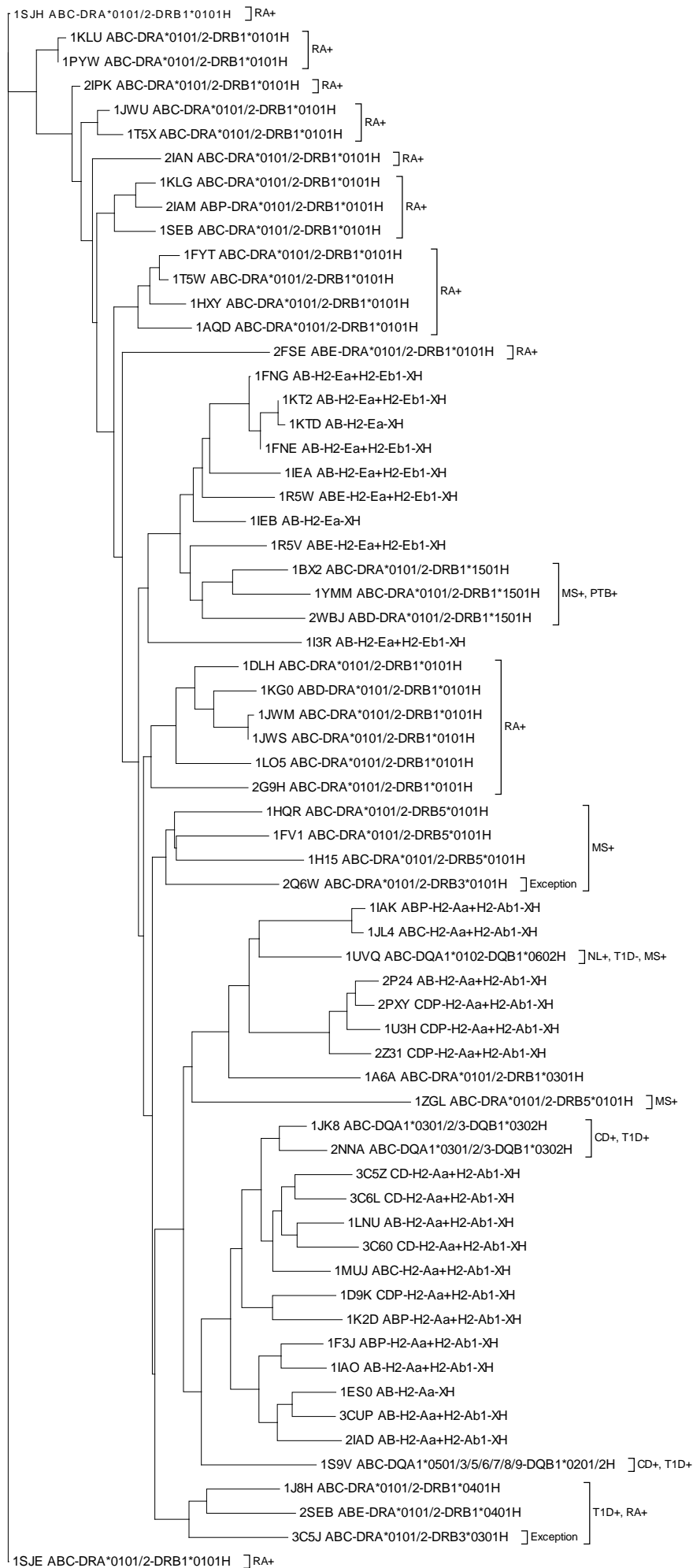

Supplement: Additional File 1 — A zip compressed archive with supplementary Figures S1-4 and Table S1. [file 1471-2105-11-S1-S55-S1.zip › Figure-S2.pdf]

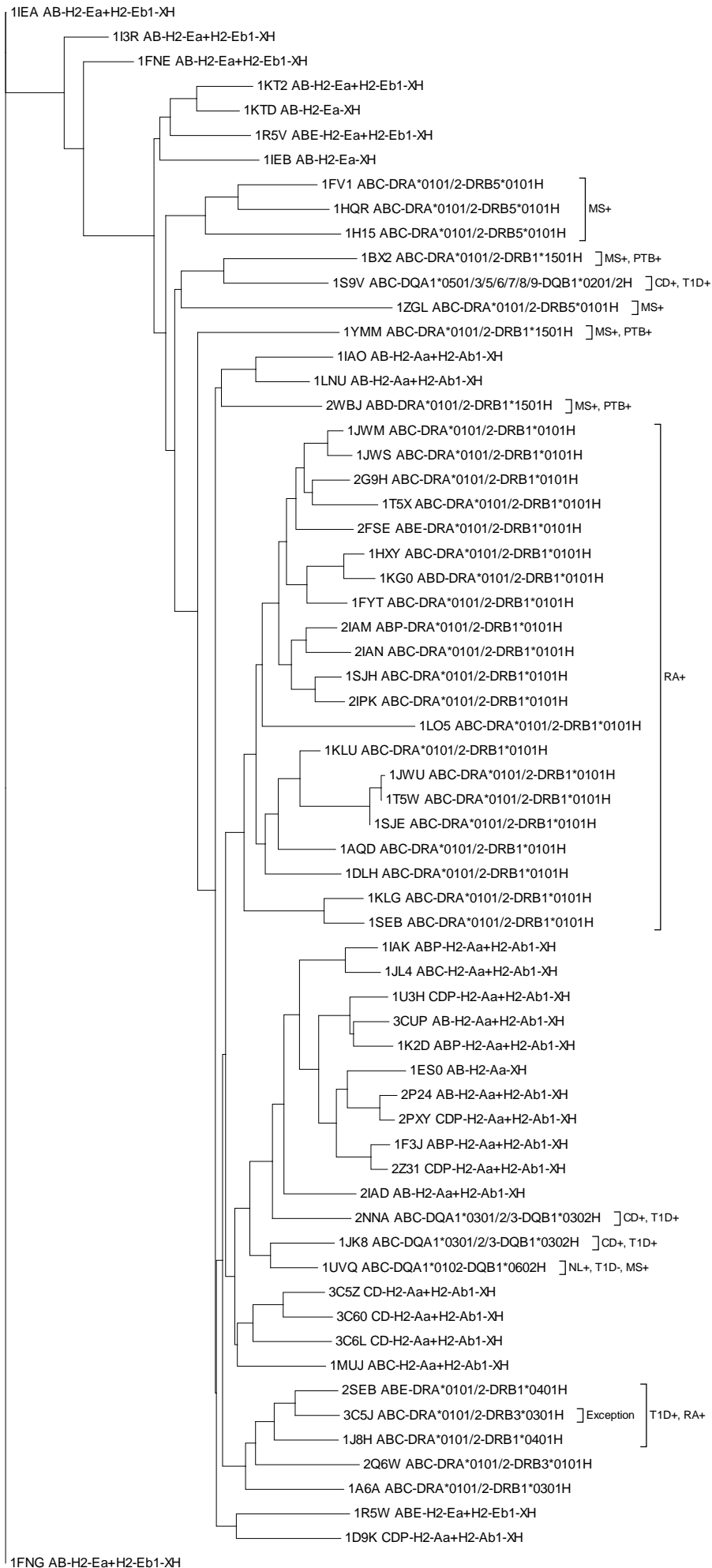

Supplement: Additional File 1 — A zip compressed archive with supplementary Figures S1-4 and Table S1. [file 1471-2105-11-S1-S55-S1.zip › Figure-S3.pdf]
